# Use of mobile learning technology among final year medical students in Kenya

**DOI:** 10.11604/pamj.2015.21.127.6185

**Published:** 2015-06-15

**Authors:** Moses Muia Masika, Gregory Barnabas Omondi, Dennis Simiyu Natembeya, Ephraim Mwatha Mugane, Kefa Ogonyo Bosire, Isaac Ongubo Kibwage

**Affiliations:** 1 Department of Medical Microbiology, School of Medicine, University of Nairobi, Kenya; 2 Partnership for Health and Development Africa, Kenya; 3 School of Pharmacy, Department of Pharmaceutics, University of Nairobi, Kenya; 4 Department of Pharmacology and Pharmacognosy, School of Pharmacy, University of Nairobi, Kenya; 5 College of Health Sciences, University of Nairobi, Kenya

**Keywords:** Smartphone, mobile-device, mobile learning, mobile application, medical education

## Abstract

**Introduction:**

Mobile phone penetration has increased exponentially over the last decade as has its application in nearly all spheres of life including health and medical education. This study aimed at assessing the use of mobile learning technology and its challenges among final year undergraduate students in the College of Health sciences, University of Nairobi.

**Methods:**

This was a cross-sectional descriptive study conducted among final year undergraduate students at the University of Nairobi, College of Health Sciences. Self-administered, anonymous questionnaires were issued to all final year students in their lecture rooms after obtaining informed consent. Data on demographics, mobile device ownership and mobile learning technology use and its challenges was collected. Data entry and analysis was done using SPSS^®^. Chi-square and t-test were used for bivariate analysis.

**Results:**

We had 292 respondents; 62% were medical students, 16% were nursing students, 13% were pharmacy students and 9% were dental surgery students. The majority were female (59%) and the average age was 24 years. Eighty eight percent (88%) of the respondents owned a smart device and nearly all of them used it for learning. 64% of the respondents used medical mobile applications. The main challenges were lack of a smart device, lack of technical know-how in accessing or using apps, sub-optimal internet access, cost of acquiring apps and limited device memory.

**Conclusion:**

Mobile learning is increasingly popular among medical students and should be leveraged in promoting access and quality of medical education.

## Introduction

Mobile phone technology has been available in one form or another for about four decades now [1, 2]. In the late nineties and early noughties, smartphones were adopted for mass usage, first in Japan then in the rest of the world [3]. The last decade has seen an exponential rise in spread and functionality of smartphones [4]. Globally, there are now over 6.8 billion mobile telephone subscribers and 70% of these are in LMICs [5]. Sub-Saharan Africa has a mobile phone penetration of about 65%. In Kenya, mobile phone penetration is about 75% [6, 7]. Currently, over half of the mobile phones being sold in Kenya are smartphones [8]. In the health sector, Mobile health (mHealth) is taking root in diverse applications such as health information, patient care and collecting data [9–12]. International technical organizations such as World Health Organization (WHO) and International Telecommunication Union (ITU) are encouraging adoption of eHealth and other Information and communication technologies (ICTs) in health facilities. One of the targets set in 2003 by the World Summit on the Information Society (WSIS) in Geneva is to connect all hospitals and health centres with ICTs by 2015. It is therefore inevitable that today′s health practitioners will have to contend with widespread technology use in their practice for which they should be well prepared. Medical schools are a good place to undertake this preparation. Smartphones are now in use in medical education for various purposes - as sources of information and reference, a guide in rounding and for enhancing problem-based learning [13–16]. The use of mobile technology in medical education is a welcome development especially because it offers a good platform for continuous self-directed learning, an important skill for all health practitioners [17, 18]. Many young doctors across the world have shown a propensity to adopt to mobile technology fast [19, 20] and some medical schools are facilitating this by offering tablets or smartphones to their medical students [21].

In Kenya, data abounds on mobile telephone use in the general population. A national survey in 2012 showed that all healthcare workers had a mobile phone with 50% of them accessing internet on their phones [22]. However, to the best of our knowledge, there′s no data on smart phone use among medical students in the country. Mobile learning (ML) breaks the limits of space and time as learning can go on anytime and anywhere by use of various devices including laptops, mobile phones, tablet computers and audio players [23]. It can be online or offline. ML offers several advantages including portability and lower cost as compared to books and desktop computers as well as augmented learning through case simulation. It also offers versatility in content delivery ranging from text, videos, audio, graphics, animation, pictures and games to interactive platforms. This makes learning more interesting and effective. ML has been applied in medical education across several contexts to serve various functions and purposes. Mobile apps are now available for a vast number of subjects ranging from basic sciences like biochemistry and anatomy to drug information, patient education and bioethics [14, 16, 24–27]. These sources of reference can be helpful for medical students during pre-rounds or ward rounds, for regular studying, and preparing for exams. Mobile applications have also been employed for transparent and objective performance assessment and evaluation of medical students by their teachers. This includes performance in Objective Structured Clinical Examinations (OSCEs) and web-based courses [28]. In addition, mobile learning provides medical students a means to self-directed learning, an important tool in the medical practice where learning is continuous and life-long [17, 18]. It also facilitates evidence-based practice by promoting access to references for medical information such as journals.

A previous study at the University of Nairobi indicated that access to new medical information was sub-optimal [29]. ML has the potential to remedy this situation. ML is mounted on several technologies. One of these is mobile applications (apps) which are programs designed to run on mobile devices. These can be native apps or mobile web browsers. Once installed, native apps are used offline though they may require intermittent internet connection for updates. Web-based applications require an internet connection to function [30, 31]. Mobile apps are run on a mobile operating system. There are several of these including Android^®^ by Google, Windows-Mobile^®^ by Microsoft, iOs^®^ by Apple, Blackberry-OS^®^ by Research in Motion, LiMo^®^ by Linux and Symbian^®^ by Accenture. More than 80% of smart phone users currently are using either Android^®^ or iOs^®^. Other platforms are Sailfish^®^, Firefox^®^, Palm-OS^®^ and Bada^®^ [30]. Operating systems can be open-source or closed-source. Open-source platforms such as Android^®^ and Limo^®^ are distributed under free licence providing universal access of the source code thus allowing modifications or use by other users at no cost [32]. Closed-source platforms are proprietary software distributed under strict license rules and the user is not allowed to modify, share or redistribute it. The source code is usually not universally available [33]. There are now tens of thousands of mobile apps built to run on various platforms. They can be obtained from application stores for a fee or at no cost. These stores include Google Play^™^, iOs App Store^™^, Windows Phone Store^™^, Blackberry World^™^, and Amazon Appstore^™^, among others. This technology is designed for various devices; usually Smartphones, Tablet computers, Personal Digital Assistants (PDAs). Each device has its unique characteristics especially in regard to processor speeds, memory size, screen size, and battery life. However, there are general characteristics that cut across the categories. Each of them is hand-held and highly portable and has technical capacity to support mobile applications, full internet browsing, blue-tooth connectivity, Wi-Fi, and several other utility features. Notably, mobile apps cannot run on basic mobile phones, including high-end ones that have touchscreen, media player, camera and Bluetooth [31].

To our knowledge, there are no studies done in Kenya or sub-Saharan Africa to explore the use of mobile learning technology among undergraduate medical students or its challenges. Nevertheless, there are dozens of studies on mHealth innovations within sub-Saharan Africa that have been completed with positive results. All indications are that use of mobile technology will continue to grow thus unless medical schools incorporate ML into medical education, there′s a looming risk of producing health care workers who are under-prepared to utilize mHealth technology fully [13]. Data on mobile phone usage in Kenya is impressive. However, we do not know the patterns of use of mobile technology for education among students including medical students. This information is key in ML content development and improving access to mobile learning for the target group and other similar groups elsewhere. This study aimed to assess use of mobile learning technology among final year undergraduate students at the University of Nairobi College of Health Sciences. Students’ preferences and challenges in accessing and using mobile learning technology were also be explored.

## Methods

This was a cross-sectional descriptive study among final year undergraduate students in the University of Nairobi, College of Health Sciences (CHS). The college has five schools, three institutes and one centre and a total of about 4200 undergraduate and postgraduate students. In 2014, the college had 491 undergraduate final year students in the Schools of Medicine (331 students), Pharmacy (70 students), Nursing Science (60 students) and Dental Surgery (31 students). This study was designed to attain a 95% confidence interval, a power of 80%, and a 5% margin of error. We used convenience sampling issuing anonymous, self-administered questionnaires to students in their lecture rooms before or after lectures after obtaining informed consent. All students available in the lecture rooms were given the opportunity to participate. We collected data on demographics, device ownership, device use and challenges to mobile learning. The questionnaire was tested among 20 nursing students before data collection began. Data was entered into SPSS^®^for cleaning and analysis. Descriptive analysis for categorical data was done using frequencies and proportions, and for continuous data using measures of central tendency. Bivariate analysis to test differences or associations was done using student t-test for numerical variables and chi-square test for categorical variables. Where expected count for any cell was less than 5, we used Fishers Exact Test, in place of Chi-square Test. This study was approved by Kenyatta National Hospital- University of Nairobi Ethics and Research Committee.

## Results

Two hundred and ninety-two final year students participated in this study. Sixty two percent were medical students, 16% were nursing students, 13% were pharmacy students and 9% were dental surgery students. The majority were female (59%) and the average age was 24 years (Table 1).

**Table 1:** Respondents’ demographic characteristics

Characteristic	School of Pharmacy	School of Nursing	School of Dental Surgery	School of Medicine	Total
**Respondents**	37 (13%)	48 (16%)	25 (9%)	182(62%)	**292**
**Sex:**					
Male	13 (36%)	19 (41%)	9 (36%)	75 (43%)	**116 (41%)**
Female	23 (64%)	27 (59%)	16 (64%)	101(57%)	**167 (59%)**
N	36	46	25	176	**283**
**Age**					
Mean	23.3	24.0	23.5	24.2	**23.9**
Standard deviation	0.944	1.15	1.25	1.05	**1.12**
Minimum	22	22	21	22	**21**
Maximum	26	28	27	28	**28**
**Fee payment mode:**					
Self-sponsored	17 (46%)	24 (51%)	10 (40%)	126(70%)	**177 (61%)**
Government-sponsored	20 (54%)	23 (49%)	15 (60%)	55 (30%)	**113 (39%)**
N	37	47	25	181	**290**
**Income (KES)**^x^^β^					
Less than 2500	12 (34%)	20 (44%)	6 (24%)	19 (12%)	**57 (21%)**
2500 – 4999	7 (20%)	11 (24%)	7 (28%)	27 (17%)	**52 (20%)**
5000 - 10,000	13 (37%)	14 (30%)	8 (32%)	69 (43%)	**104 (39%)**
Over 10,000	3 (9%)	1 (2%)	4 (16%)	45 (28%)	**53 (20%)**
N	35	46	25	160	**266**

x **KES = Kenya shillings**

β **One United States Dollar = KES. 85.00**

**Figure 1:**
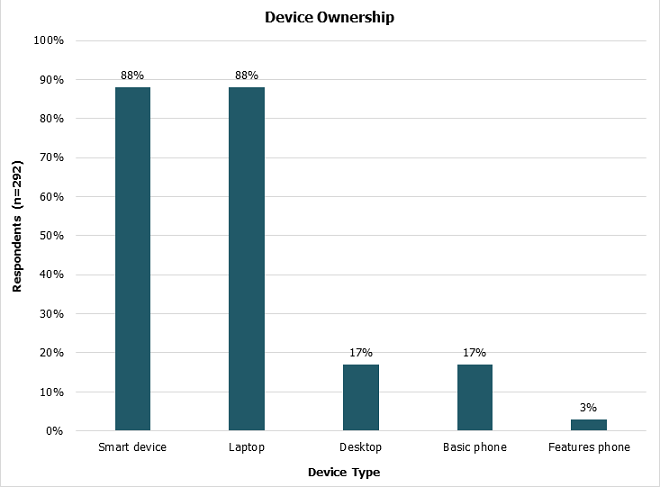
Device ownership

**Device ownership:** Most of the respondents owned a smart device (88%, n = 258/292). This was either a tablet or smart phone (including iPhones, and Blackberries). A similar number of students owned laptops too. Eighty percent of the respondents owned both a laptop and a smart device (n = 235/292) (Figure 1). Students who had a monthly income above KES. 5000 (USD. 55) were more likely to own a smart device (p = 0.047). Students who did not own a laptop were less likely to own a smart device (p < 0.001). Reasons offered for not owning a smart device were cost (65%), preference (20%), loss or theft (15%). The most popular device makes were Samsung (46%), Tecno (12%) and Apple (10%). Other brands were Nokia (9%), Sony (8%), Huawei (7%), Alcatel (5%) and LG (5%), each of which were owned by under 10% of the respondents (n = 232).

**Device platform:** The most popular mobile operating systems for smart devices were Android (85%), Windows Mobile (10%) and iOs (Apple -10%). Other platforms were used by 2% of the respondents (n = 250).

**Figure 2:**
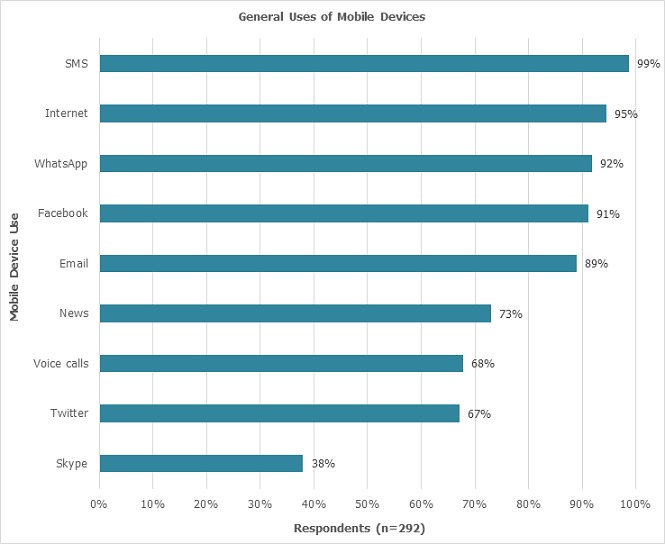
General uses of mobile devices by medical students at the University of Nairobi

**General Use of smart device:** Nearly all participants used their smart devices for short messaging service (SMS), internet access, social media and email (Figure 2).

**Figure 3:**
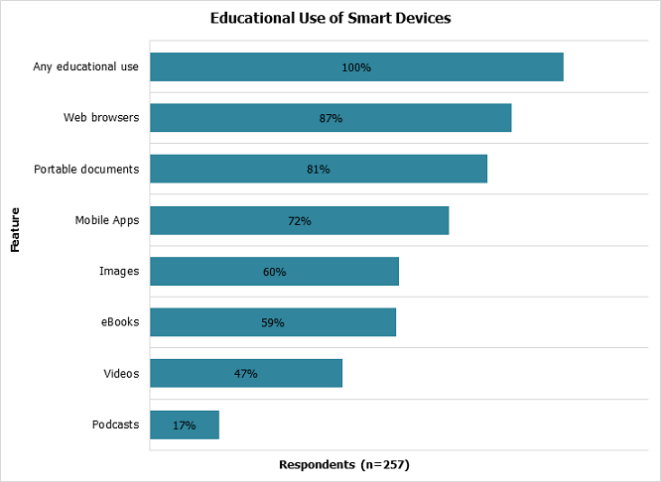
Educational use of smart devices by medical students at the University of Nairobi

**Educational use:** Virtually all respondents who owned a smart device reported using it for learning (n = 257/258). The major educational features accessed on smart devices were web browsers (87%), portable documents (81%), mobile applications (72%), images (60%) and eBooks (59%). Less commonly accessed materials were videos (47%) and podcasts (17%) (Figure 3). Smart device holders accessed learning materials for several purposes: Regular study (85%), revising for exams (74%), taking notes or images (62%) and accessing research journals (46%).

**Mobile applications:** Sixty four percent of all respondents had medical mobile applications (n = 186/292). 72% of smart device holders (186/258) listed at least one app they had used on their device (an average of 1.5 apps, standard deviation = 1.56). 65% of smart device holders reported having 1-5 medical mobile applications on their device. The most commonly listed apps were Medscape^™^ (66%), drug index apps (9%), and medical dictionaries (7%). The most accessed app types were disease management apps (88% of respondents), procedure guides (88%), and medical dictionaries (87%). Other commonly accessed apps were for laboratory reference (81%), drug reference/index (73%) and medical calculators (31%) (Table 2).

**Table 2:** Frequency of mobile application use by type

Application category	Never used	Rarely used	Occasionally used	Often used	Constantly used	Ever used	n
Disease management	12%	7%	25%	43%	13%	88%	239
Procedure guide	12%	12%	36%	33%	7%	88%	242
Medical dictionaries	13%	15%	30%	30%	12%	87%	243
Lab reference	19%	13%	28%	31%	10%	81%	231
Drug index	27%	17%	25%	24%	6%	73%	240
Medical calculators	31%	20%	28%	15%	7%	69%	234

This table shows how frequently University of Nairobi medical students use different Mobile Application types.

**Payment for medical apps:** Fifteen percent (15%) of the respondents had ever paid for a medical app. 43% would be willing to pay for one in the future (n = 292). There was no association between willingness to pay and the income of the respondent.

**Proficiency:** The majority of respondents reported moderate proficiency in mobile application use. Half of the respondents had installed several apps to test the experience. A few (1%), reported having developed their own app (Table 3).

**Table 3:** Medical students’ proficiency in mobile application use

Statement	Respondents	Percent
I do not use mobile apps	10	4%
I use a few factory installed apps	18	6%
I have installed a few apps that I have seen or heard about	106	38%
I have installed many apps to test the utility	143	51%
I have developed my own apps	3	1%

This table shows how medical students self-rated their skills in use of Mobile Applications

**Accessing Medical journals:** Eighty six percent of all respondents (n = 228/266) had ever accessed a medical journal. Respondents who owned a smart device were more likely to access medical journals though the association was not statistically significant (p = 0.058).

**Cellular internet connection:** The most popular cellular internet provider among the respondents was Safaricom (85%). Airtel Kenya had 15%, Orange 7% and Yu Mobile 2%.

**Potentially harmful use of smart devices:** Nearly a third (29%) of the respondents had ever used a mobile device while driving, 74% had used a device during sleep breaks and 85% during lectures (Table 4).

**Table 4:** Potentially harmful use of mobile devices by medical students at the university of Nairobi

Device Use	Never	Rarely	Occasionally	Often	Always
During lectures	15%	26%	35%	19%	5%
During sleep breaks	26%	14%	22%	22%	16%
While driving	71%	13%	11%	3%	2%

**Limitations of mobile application use:** Several limitations to the use of mobile applications were cited- 29% of the respondents did not know how to download apps, 28% had devices that could not support installation of new apps, 27% had limited internet access and 23% did not know which apps to download. A few felt that apps were expensive (16%) or downloading apps was expensive (5%). Ten percent reported limited device memory as a hindrance.

**Desired information:** Respondents reported that they would like to access medical journals on their devices (41%), 20% desired to have free access to ‘for-pay’ apps, 14% desired to have apps developed for Kenya while a similar number desired to access drug index apps on their device. Some students desired to access their exam results or transcripts (8%), to access clinical guidelines (8%), lecture notes (3%) or Free Wireless internet connection on their devices (3%).

## Discussion

This study aimed at assessing the use of mobile learning technology by final year undergraduate students at the College of Health Sciences, University of Nairobi as well as exploring the challenges that impede adoption of mobile learning technology in the target population. We found that most of the students owned smart devices, a majority of which run on the Android^™^platform. Nearly all students who owned a smart device used it for learning. The main educational uses were regular study, revising for exams, taking notes or images and accessing research journals. About three quarters of the students with smart devices were using medical mobile applications. These were mainly disease management apps, procedure guides, medical dictionaries, laboratory references, drug indexes and medical calculators. The main challenges were lack of a smart device, lack of technical know-how in accessing or using apps, lack of internet access, cost of acquiring apps and limited device memory. Our findings show that smart device ownership among medical students is higher than in the general Kenyan population (88% versus 51%) [8]. As their cost goes down, smartphones are becoming increasingly popular among university students. Our findings compare with studies from South Korea [34] and Saudi Arabia [35] which showed that nearly all university students own a smart phone. In the United Kingdom (UK) [20] and the United States (USA) [36], about 80% of medical students own a smart phone. Mobile learning, though relatively a new concept, is highly popular among medical students in Kenya. This reflects findings from work done in other countries such as the UK, [20] and USA [37]. Limitations to adoption of mobile learning technology described in this study such as cost and sub-optimal internet connection are similar to those depicted elsewhere. Some limitations are technical such as short battery life, small screen size, and application incompatibility across various operating systems. Cost, privacy and security concerns also limit the use of mobile technology as does the rapid growth that often leads to gadget obsolescence as new apps may be incompatible with older devices. Sub-optimal penetration of broadband internet connectivity is also a limitation [31]. Lack of awareness of available applications or how to access and use them is also a hindrance [15, 20, 38]. ML also requires institutional readiness and human and infrastructural resources that may not always be available in low and middle income countries [39]. A limitation for this study is that technological jargon is not always clear to everyone. Although every effort was made to make the questionnaire as simple and unambiguous as possible, it is possible that some respondents may have misunderstood some of the questions. In this regard, a questionnaire administered by trained personnel, in place of self-administered questionnaires may have offered more accurate responses. This was not possible due to resource limitations.

## Conclusion

This study shows that mobile learning is popular among medical students. It also shows key challenges to the adoption of ML in medical education. This information should be useful to modern-day medical educators that are seeking to exploit mobile technology to improve access to and quality of medical education. Mobile learning is likely to increase among medical students. There's need to apply more effort in developing mobile technologies that fit the needs of students. These may include local national clinical guidelines, national drug index/formulary, university organization and administration information such as timetables, exam results and lecture notes among others. University campuses should also provide students with easy-to-connect, yet secure, free internet access.
